# Reproducibility and Accuracy of a New Method for Measuring the Range of Dart-Throwing Motion

**DOI:** 10.1016/j.jhsg.2025.01.013

**Published:** 2025-02-22

**Authors:** Masahiro Mitsukane, Akino Aoki, Tomohiro Kakehi, Ryu Kobayashi, Naotoshi Kimura

**Affiliations:** ∗ Department of Occupational Therapy School of Health Sciences at Narita, International University of Health and Welfare, Narita City, Chiba, Japan; † Department of Physical Therapy, School of Health Sciences at Narita, International University of Health and Welfare, Narita City, Chiba, Japan

**Keywords:** Accuracy, Dart throw, Range of motion, Reproducibility, Wrist

## Abstract

**Purpose:**

To evaluate the reproducibility and the accuracy of our technique to measure the range of dart-throwing motion.

**Methods:**

Two raters measured the range of dart-throwing motion of 42 healthy participants. The participants performed a simulated hammering action with a wooden mallet, and the inclination angle of the mallet on the vertical plane was measured using an attached bubble inclinometer at the maximal position of radial extension and ulnar flexion. The sum of these angles was defined as the range of the dart-throwing motion. Each rater performed three measurement trials for each participant. To determine inter-rater reproducibility, intra-class correlation coefficients were calculated for the value of one trial, mean value of two trials, and mean value of three trials. In the first test session, wrist kinematics during measurement was recorded simultaneously using a three-dimensional optical motion capture system.

**Results:**

Intra-class correlation coefficients for the dominant and nondominant sides ranged from 0.67 to 0.75 and 0.68 to 0.79, respectively. The reproducibility of the measurements was improved by adopting the mean value as the number of repetitions of the measurements increased. Bland–Altman analysis revealed that our measurement contained a proportional bias of 30.7% to 35.9% compared with the values of the motion capture analysis as the gold standard.

**Conclusions:**

The reproducibility of the measurements was either good or moderate. The revealed biases can provide valuable data for estimating the true range of wrist motion.

**Clinical relevance:**

Our technique would be useful for reliable measurement of the range of dart-throwing motion, as it is easy to perform repeated measurements. Our method avoids observer bias even by a single examiner and can be carried out with readily available materials.

In many daily activities involving tool handling, the wrist joint moves along the path from radial extension to ulnar flexion.^1^, ^2^, ^3^ As this motion is symbolized in the act of throwing a dart, Palmer et al^2^ named it the dart-throwing motion (DTM) and described it as a functional and natural motion of the wrist. In 2007, the International Federation of Societies for Surgery of the Hand formally defined DTM as a functional oblique motion of the wrist joint occurring from radial extension to ulnar flexion.^4^

Studies examining the relationships between the range of wrist motion and Disabilities of the Arm, Shoulder, and Hand (DASH) scores revealed that the range of DTM was the only measure that correlated with the disability score.^5^^,^^6^ The DASH outcome measure is a self-report questionnaire designed to measure physical function and symptoms and describes disability in upper-limb disorders.^7^ These studies suggest that the DTM range is a sensitive proxy for disabilities with respect to activities of daily living.

The DTM range is expected to become more widespread as a highly useful outcome; however, a standardized method for its measurement has not yet been established. This may be because no measurement method that is both reproducible and sufficiently simple to withstand clinical use has yet been presented. In 2012, Kasubuchi et al^8^^,^^9^ developed a specialized device for measuring the DTM range and reported that highly reproducible measurements could be made using this device. Their measurement device has dimensions of 170 mm (W) × 482 mm (D) × 150 mm (H) and weighs 1.4 kg. Currently, this dedicated device is not commonly used because it is not readily available. In 2013, Bugden^10^ proposed a method for measuring the DTM range using a conventional manual goniometer. However, it has been noted that the identifications of measurement axes recommended by Bugden are difficult on the actual participant’s body.^11^ We previously proposed a novel technique that does not rely on the body of the participant for these axes and found that the reliability was good to moderate.^12^ In addition, this technique is convenient because it employs readily available materials.

In the current study, we made a minor modification to our technique to reduce the measurement error. Therefore, this study aimed to examine the reproducibility of our modified technique, and the accuracy of the measurement was verified by evaluating its agreement with optical motion capture measures as the gold standard.

## Materials and Methods

### Study protocol

We adopted a test–retest design to assess inter-rater reproducibility, in which the DTM range was measured by two raters at an interval of several days for each participant. The assignment order of the raters was randomized for each participant. In the first test, before the interval, wrist kinematics during the measurement of the DTM range was simultaneously analyzed using optical motion capture.

### Participants

Forty-two healthy participants (24 males and 18 females) with no history of orthopedic disease that could cause any limitation in wrist motion participated in the study. Mean age, height, and weight were 20.2 ± 1.3 years (range, 18–22 years), 165.4 ± 8.4 cm (range, 148–183 cm), and 58.1 ± 11.6 kg (range, 40–90 kg), respectively. A total of 38 participants were right-handed, and four were left-handed.

Two occupational therapists with >10 years of clinical experience served as raters. They received an adequate explanation of the measurement procedure and practiced for approximately 1 hour to ensure mastery of the skills before the experiment.

Before enrollment, the participants were given an information sheet, and written informed consent was obtained for their participation. Ethical approval was granted by the Research Ethics Committee of the International University of Health and Welfare (approval number 23-Im-014-2).

### DTM measurement

**Figure 1:**
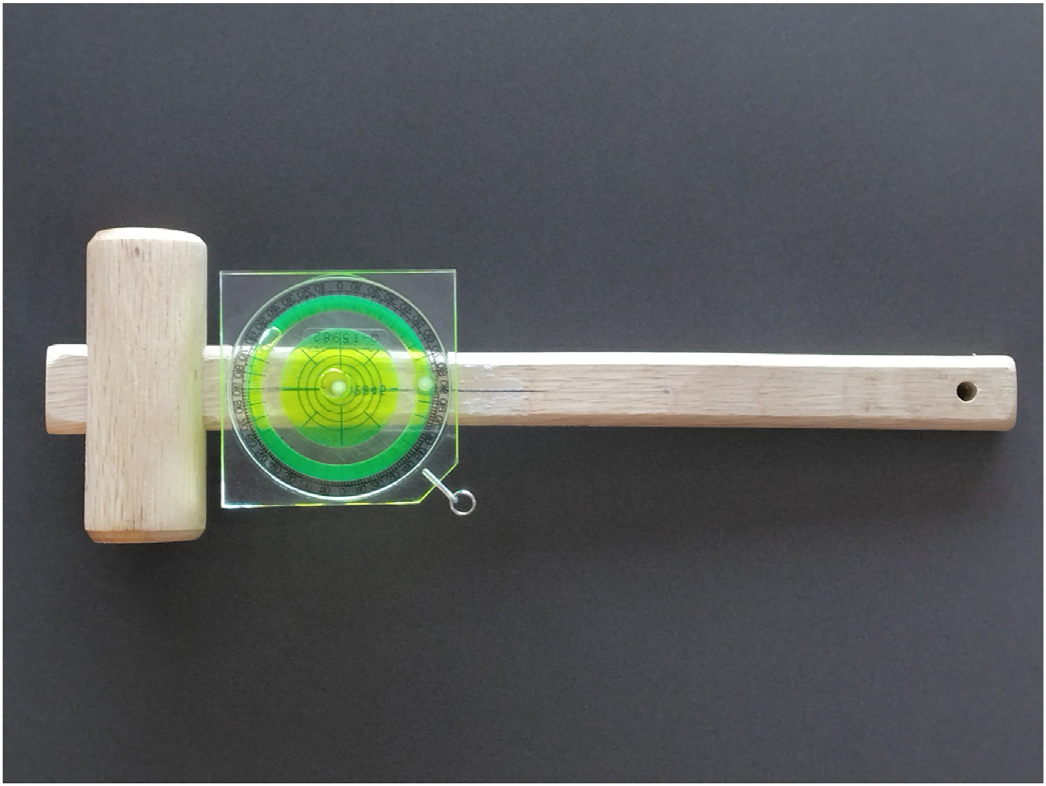
Measuring instrument.

A measuring instrument consisting of a wooden mallet and bubble inclinometer was used to measure the DTM range (Fig.1). The bubble inclinometer was mounted such that its horizontal and vertical scales were parallel to the long axis of the mallet’s shaft and the striking direction of the tip, respectively. The mallet was 30 cm long, and the total weight of the instrument was 230 g. The center of gravity was located 20 cm distal to the proximal edge of the grip. The cross-sectional shape of the grip was rectangular with major and minor axes of 2.3 and 1.2 cm, respectively, with beveled corners.

**Figure 2:**
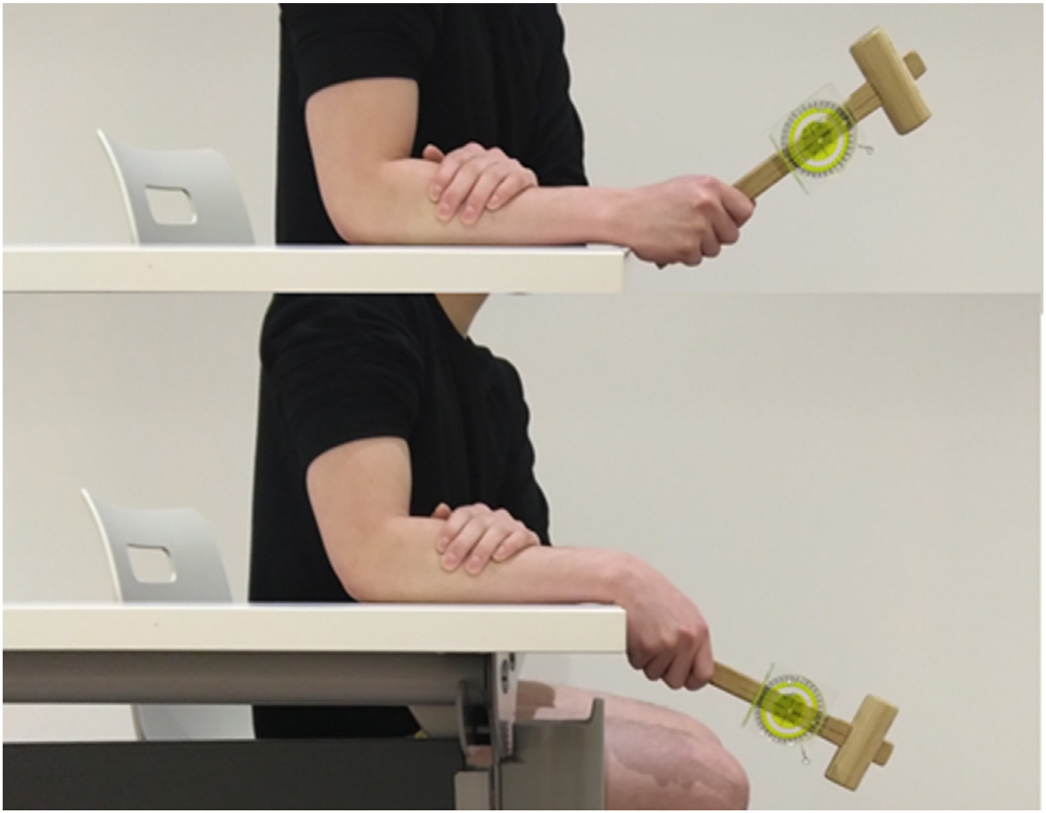
Measuring the DTM.

The participants were seated on a chair. The forearm to be tested was placed on a table and held against it by the participants’ opposite hand to restrict the motion of the elbow joint. The forearm position with respect to pronation and supination was left to the participant’s discretion. The participants held the measuring instrument and performed a simulated hammering action in the vertical direction. While holding the instrument, the grasping position and finger posture were left to the participant’s discretion. Once these values were determined, the participants were not permitted to change their position or posture during the measurement. The angle between the long axis of the mallet shaft and the horizontal plane was measured using the bubble inclinometer at the maximal position of radial extension and ulnar flexion. The upward and downward motions beyond the horizontal plane were expressed as positive angles for radial extension and ulnar flexion and were expressed as negative angles when the motion did not proceed beyond the horizontal plane. The sum of the radial extension and ulnar flexion angles was defined as the DTM range (Fig. 2). The minimum measurement unit was 5°.

From the maximum radial extension position on the DTM plane, the participants initiated the repetition of the hammering action on the cue by the rater, stopped at the maximum position of the third downward motion (ulnar flexion), and remained still. The inclination angle of the instrument was recorded simultaneously with motion capture markings. Subsequently, the participants started the repetition of the hammering action on the cue by the rater, stopped at the maximum position of the third upward motion (radial extension), and remained still. The inclination angle of the instrument was recorded simultaneously with motion capture markings. When performing the simulated hammering, the reciprocating time required for the combination of one upward and one downward movement was set at approximately 1 second. Three measurement trials were performed for each test, with a break of a few tens of seconds between trials. We provided a break time to allow the participants to regrip the measuring instrument for the next trial. Three trials were first performed on the dominant hand, followed by three trials on the nondominant hand.

In the orientation of the participants, the following precautions were explained: forearms should be firmly fixed, especially pronation-supination motion should be consciously avoided; hammering action should be performed in the vertical direction; although the measuring instrument can be grasped in any way, the finger position should not be changed during the hammering action; and the instrument should not be allowed to move in the hand. After orientation, we had the participants practice this exercise and confirmed that these precautions had been followed; we then began the measurement.

### Optical motion capture

**Figure 3:**
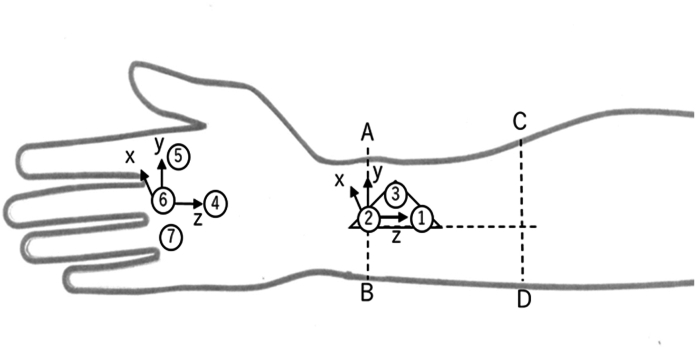
Marker placement and local coordinate system.

Vicon Motion Capture Systems (Vicon MX) were used for three-dimensional motion analysis. To record the global wrist motion, seven light reflective markers of 5.0 mm diameter were attached to the dorsum of the distal forearm and the hand of the participant, as specified in the study by Li et al^13^ (Fig. 3). An isosceles triangular (base 40 mm, height 20 mm) plastic plate, in which markers 1, 2, and 3 were placed at each apex, was attached such that the base of the plate was aligned with the median line of the forearm. Lines AB and CD in Figure 3 are vertical lines drawn while the participants placed their forearm on a table, positioning the palm aspect perpendicularly. Line AB is drawn approximately at the center of the forearm, and line CD is drawn across the ulnar styloid process. The line connecting the midpoints of AB and CD is defined as the median line of the forearm. Markers 5, 6, and 7 were attached to the heads of the second, third, and fourth metacarpals, respectively, whereas marker 4 was attached to the middle of the third metacarpal.

For the data processing, local coordinate systems were created for the rigid bodies of the forearm and hand. The z axis of the forearm frame pointed proximally from marker 2 to marker 1, the x axis was in the dorsal direction and perpendicular to the plane formed by markers 1–3, and the y axis was in the radial direction. The z axis of the hand frame was proximal to markers 6–4. The x axis was in the dorsal direction and perpendicular to the plane formed by markers 4, 5, and 7. The y axis was in the radial direction perpendicular to both the z and x axes. When the two z axes were parallel, the position of the wrist joint was defined as neutral. The roll and pitch of the hand local coordinate system relative to the forearm local coordinate system were recorded. The range of wrist motion during the simulated hammering action and the position of the performed DTM plane relative to the sagittal anatomical flexion-extension motion plane were also recorded. Prior to the analysis, we assumed that the hammering action was carried out on the motion plane passing through the two wrist positions that had been marked during motion capture; that is, it was a linear action.

### Statistical analysis

Intra-class correlation coefficients (ICC2, 1) were used to examine inter-rater reproducibility. The ICC was calculated for the measurement of the first trial only, the average of the first and second trials, and the average of three trials. Reproducibility was defined as poor (ICC < 0.50), moderate (ICC 0.50–0.75), or good (ICC > 0.75) using previously established criteria.^14^

To verify the accuracy of the measurement, Bland–Altman analysis was performed using paired data from the measuring instrument and motion capture analysis.^15^ Calculations included 95% confidence intervals for the mean difference between the two paired measures, regression between the difference and mean of the two paired measures, and 95% limits of agreement (LOA). When 0 did not lie within the 95% confidence interval for the mean difference between two paired measures, a fixed bias was considered to exist in our technique relative to the gold standard. When the regression between the difference and mean of the two paired measures was significant, a proportional bias was considered. The 95% LOA was calculated using the equation: 95% LOA = mean difference ± 1.96× SD_d_ (SD_d_ = standard deviation of difference). When a proportional bias was found, the variable of the difference between the two paired measures was replaced with its ratio to the mean of those measures to calculate the bias and 95% LOA.

## Results

Table 1 presents the descriptive statistics of the measures. Motion capture analysis indicated that the mean plane of the simulated hammering action was approximately 30° to the sagittal plane of the forearm, inclined from a position of radial extension to ulnar flexion, and offset by 25.8° to 31.8° in extension from the anatomically neutral wrist position.

**Table 1:** Descriptive Statistics for the Measurements

Adopted Values	DTM Range Measured With the Measuring Instrument			Motion Capture Analysis		
	Values by Rater A	Values by Rater B	Values at the First Test	DTM Range	Motion Plane Position	
					Slope∗ (Degree)	Offset† (Degree)
First						
Dominant	76.9 ± 17.1 (45.0–115.0)	74.5 ± 18.3 (35.0–100.0)	72.7 ± 17.1 (35.0–110.0)	50.8 ± 13.1 (22.5–78.6)	32.5 ± 22.3 (−20.7 to 72.4)	25.8 ± 40.4 (−81.1 to 88.4)
Nondominant	79.8 ± 17.1 (50.0–120.0)	79.2 ± 21.0 (35.0–115.0)	76.3 ± 19.8 (35.0–115.0)	56.2 ± 15.3 (22.1–84.1)	36.6 ± 19.6 (15.6–69.2)	35.2 ± 25.6 (–67.0 to 81.7)
First–second						
Dominant	78.6 ± 17.4 (47.5–122.5)	75.8 ± 18.8 (32.5–102.5)	74.2 ± 17.3 (32.5–112.5)	52.1 ± 12.9 (22.1–77.6)	32.9 ± 19.8 (−17.9 to 57.1)	29.1 ± 28.2 (−75.0 to 80.0)
Nondominant	80.2 ± 16.9 (47.5–120.0)	79.3 ± 20.1 (35.0–112.5)	76.8 ± 18.8 (35.0–112.5)	56.1 ± 14.4 (22.6–86.8)	36.9 ± 20.0 (−12.0 to 75.3)	33.7 ± 25.2 (−71.3 to 81.6)
First–third						
Dominant	79.0 ± 17.1 (45.0–118.3)	76.5 ± 19.1 (31.7–105.0)	75.2 ± 17.7 (31.7–115.0)	52.6 ± 12.8 (22.5–78.4)	33.2 ± 18.9 (−16.8 to 56.6)	31.8 ± 26.5 (−76.8 to 77.1)
Nondominant	80.6 ± 17.4 (43.3–120.0)	80.1 ± 19.8 (36.7–113.3)	77.6 ± 18.5 (36.7–113.3)	56.8 ± 13.9 (24.4–84.6)	37.9 ± 18.8 (−8.6 to 75.2)	32.8 ± 25.3 (−76.4 to 77.1)

Data are indicated in mean ± standard deviation (range). First, value of the first trial; First–second, mean value of the first and second trials; First–third, mean value of the first to the third trial.

∗ The slope of the performed motion path with respect to the sagittal anatomical flexion-extension motion plane on the transverse plane. The minus value indicates that the wrist moves along the path from ulnar extension to radial flexion.

† Offset in extension from the anatomical neutral wrist position. The minus value indicates the offset in flexion from the anatomical neutral wrist position.

Table 2 presents the reproducibility of the measurements. The mean time between the first test and the retest was 3.0 ± 2.2 days (range, 1–8). The ICC for the dominant and nondominant sides ranged from 0.67 to 0.75 and 0.68 to 0.79, respectively. The reproducibility of the measurements was improved by adopting the mean value as the number of repetitions of the measurements increased.

**Table 2:** Reproducibility of Measurements

Adopted Values	Dominant	Nondominant
First	0.67 (0.47–0.81)	0.68 (0.47–0.81)
First–second	0.71 (0.52–0.83)	0.77 (0.60 –0.87)
First–third	0.75 (0.58–0.86)	0.79 (0.65–0.88)

Values are intra-class correlation coefficients (ICC 2,1) and associated 95% confidence intervals. First, value of the first trial; first–second, mean value of the first and the second trial; first–third, mean value of the first to the third trial.

**Figure 4:**
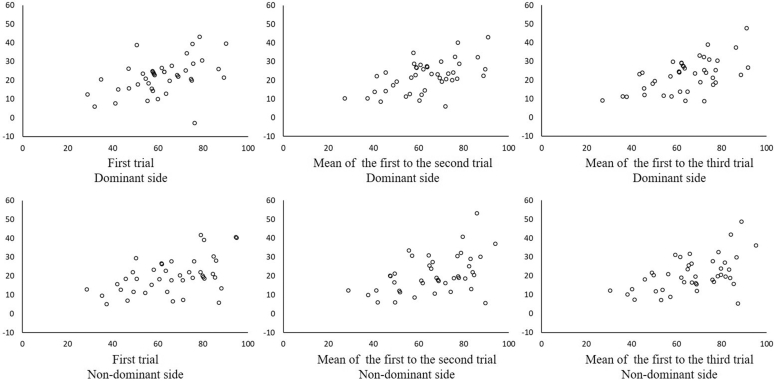
Bland–Altman plots. The upper three diagrams show the Bland–Altman plots for the dominant side and the bottom three show the Bland–Altman plot for the nondominant side. The results are shown when we adopted the value of the first trial, the mean value of the first to the second trial, and the mean value of the first to the third trial, from left to right respectively. The x axes represent the mean of the two values (degrees), and the y axes represent the difference between the two values (degrees).

Figure 4 shows the Bland–Altman plots, and the results of the analysis revealed that our measures contained a proportional bias ranging from 30.7% to 35.9% compared with the gold standard (Table 3).

**Table 3:** Results of the Bland–Altman Analysis

Adopted Values	Mean Difference (Degree)	95% CI (Degree)	γ	*P* Value of γ	Proportional Bias	95% LOA
First						
Dominant	21.9	19.0–24.8	0.45	.003	35.9 (%)	9.4–62.4 (%)
Nondominant	20.1	17.2–23.1	0.5	<.001	30.7 (%)	7.2–54.2 (%)
First–second						
Dominant	22.1	19.5–24.7	0.54	<.001	35.2 (%)	13.2–57.2 (%)
Nondominant	20.8	17.6–23.9	0.46	.002	31.4 (%)	6.2–56.6 (%)
First–third						
Dominant	22.6	19.9–25.4	0.57	<.001	35.5 (%)	14.2–56.9 (%)
Nondominant	20.8	17.9–23.7	0.51	<.001	31.1 (%)	9.1–53.1 (%)

First, value of the first trial; first–second, mean value of the first and the second trials; first–third, mean value of the first to the third trial. CI, confidence interval for the mean difference; γ, correlation coefficient.

## Discussion

We have previously proposed a novel technique for measuring the DTM range.^12^ In this method, the participants were asked to hold a cylindrical rod or wooden mallet and perform a simulated hammering action. The range of motion of the object on the vertical plane measured with the manual goniometer was defined as the DTM range. The movable axis was the long axis of the object, and the stationary axis was the string of plumbs attached to the object. In general, the reproducibility of the measurements was improved by repeating the measurements and using the mean values. When an examiner alone evaluates the range of motion using a manual goniometer, it is difficult to repeat measurements to avoid observer bias. In this study, we made modifications such that the range of the measuring instrument was measured directly with an attached inclinometer, rather than by goniometry. Although the bubble inclinometer induces a new problem of inaccurate measurements when the measurement plane is not parallel to the vertical plane (owing to rotation around the long axis of the mallet handle), it facilitates repeated measurements, avoids observer bias, and completely eliminates measurement errors that can occur during goniometer operation.

Recently, several authors reported the reliability of goniometric measurements of the DTM range. Parish et al^16^ measured the DTM range in patients with hand or wrist injuries while adhering to the method proposed by Bugden.^10^ The participant was asked to perform simulated hammering while holding a real hummer, and goniometry was performed along the body of the participant. They reported that the ICC of one trial was 0.83 for radial extension and 0.70 for ulnar flexion. The ICC tended to be higher when there was large variability in the measured values among the participants. Therefore, we believe that further verification is required to confirm whether similar results can be obtained even when targeting healthy participants. Meanwhile, as for healthy participants, Cliff and Rust^17^ reported high ICC values of 0.83 and 0.81 for radial extension and ulnar flexion, respectively, for the inter-rater measurements. In their method, the participant was asked to perform a simulated dart throw, and goniometry was performed along the body of the participant. The value used for verification was the average of three trials, and a recording secretary was provided separately. In the current study, our technique appeared to withstand clinical use and exhibited good to moderate reproducibility. For clinical use, taking advantage of the ease of repeated measurements, we thought that it would be desirable to repeat a couple of trials and adopt an average value to obtain higher reproducibility.

Although our method is reliable and convenient, it has the following limitations. First, because the kinematic 0° cannot be defined, measurements of the range of radial extension and ulnar flexion are not regarded theoretically as true values. Second, our technique targets only the active range of motion. Third, those who have difficulty in grasping the measuring instrument may not be examined.

According to a study using optical motion capture in nine healthy male volunteers, the DTM plane during hammering a nail showed a mean inclination of 42° ± 9° with respect to the sagittal anatomical flexion-extension motion plane.^3^ In another study using computed tomography in thirteen healthy participants, the DTM plane of simulated hammering showed a mean inclination of 41° ± 3° with respect to the anatomical flexion-extension sagittal plane and was offset by an average of 36° ± 8° in extension at 0° of radial-ulnar deviation.^18^ The number of participants in the current study was larger than those in these reports, and the position of the DTM plane was slightly different from the results of these studies. This means that there was greater individual variation in the path of the DTM than previously recognized. Using our motion capture results as the gold standard, this study revealed that proportional bias was included in the value of our technique. These findings could be valuable references for estimating the true range of wrist motion based on the measurements of our technique. It seemed that the proportional bias shown was primarily caused by the difference in orientation between the third metacarpal axis, which was defined as the movable axis of the motion capture and the long axis of the measuring instrument where the inclinometer was attached; however, the current study did not provide satisfactory evidence to confirm this. Regarding the overall measurement error, which is a combination of both systematic and random errors, we considered that the sway of the measuring instrument in the hand, misalignment of the measurement plane of the inclinometer mentioned above, and misalignment of the motion path of the measuring tool to the vertical plane could be errors. However, we were unable to confirm how these factors were affected.

In our previous report, we investigated how the difference in tools held by the participant affected the measurement.^12^ The objects were a wooden mallet and two types of 30 cm long cylindrical rods of different thicknesses and weights. The results showed no statistically significant differences in the measures between the tools. Therefore, we suggest that any object considered applicable to hammering based on common sense can be used in our technique. In the standardization process, it is better to choose what is readily available. When comparing a wooden mallet and cylindrical rod, the latter is considered more appropriate. However, we chose the former in this study because the wooden mallet would make it easier for the participant to define the rotational position of the grip handle because of the presence of a striking part; thus, the parallelism between the measurement plane of the bubble inclinometer and the vertical plane could be easily maintained. If we could use an inclinometer that does not require this parallelism, we would be able to measure with the same or less measurement error than that in the current study, even with a simple cylindrical rod instead of a wooden mallet.

## Conflicts of Interest

No benefits in any form have been received or will be received related directly to this article.

## References

[1] Capener N. (1956). The hand in surgery. J Bone Joint Surg Br.

[2] Palmer A.K., Werner F.W., Murphy D., Glisson R. (1985). Functional wrist motion: a biomechanical study. J Hand Surg Am.

[3] Brigstocke G.H., Hearnden A., Holt C., Whatling G. (2014). In-vivo confirmation of the use of the dart thrower’s motion during activities of daily living. J Hand Surg Eur Vol.

[4] Moritomo H., Apergis E.P., Herzberg G., Werner F.W., Wolfe S.W., Garcia-Elias M. (2007). 2007 IFSSH committee report of wrist biomechanics committee: biomechanics of the so-called dart throwing motion of the wrist. J Hand Surg Am.

[5] Kasubuchi K., Hukumoto T., Dohi Y. (2013). A relationship between dart-throwing motion plane ROM and the DASH score after distal radius fracture [Japanese]. J Jpn Phys Ther Assoc.

[6] Kasubuchi K., Dohi Y., Ono H. (2013). A relationship between dart-throwing motion plane ROM and the DASH score after distal radius fracture: analysis of change over time [Japanese]. J Jpn Soc Surg Hand.

[7] Imaeda T., Toh S., Nakao Y. (2005). Validation of the Japanese Society for Surgery of the Hand version of the Disability of the Arm, Shoulder, and Hand questionnaire. J Orthop Sci.

[8] Kasubuchi K., Dohi Y., Hujita H. (2012). Development of a goniometer to measure the range of motion in the dart-throwing motion plane [Japanese]. Jpn J Clin Biomech.

[9] Kasubuchi K., Dohi Y., Fujita H., Fukumoto T. (2019). Reliability and responsiveness of a goniometric device for measuring the range of motion in the dart-throwing motion plane. Physiother Theory Pract.

[10] Bugden B. (2013). A proposed method of goniometric measurement of the dart-throwers motion. J Hand Ther.

[11] Mitsukane M., Tanabe H., Sugama K., Suzuki Y., Tsurumi T. (2019). Test-retest reliability of goniometric measurements of the range of dart-throwing motion. J Phys Ther Sci.

[12] Mitsukane M., Sugama K., Suzuki Y., Tanabe H., Tsurumi T. (2020). Proposal for a method to measure the range of dart-throwing motion. J Hand Surg Glob Online.

[13] Li Z.M., Kuxhaus L., Fisk J.A., Christophel T.H. (2005). Coupling between wrist flexion-extension and radial-ulnar deviation. Clin Biomech (Bristol).

[14] Portney L.G., Watkins M.P. (2009).

[15] Bland J.M., Altman D.G. (1986). Statistical methods for assessing agreement between two methods of clinical measurement. Lancet.

[16] Parish M., Bugden B., Liu K.P.Y. (2018). Psychometric properties of the goniometric assessment of the dart-thrower’s motion. Hand Ther.

[17] Cliff N.J., Rust P.A. (2016). A study to investigate the intra-rater and inter-rater reliability of goniometric measurements of dart throwers motion of asymptomatic wrists. HandTher.

[18] Leventhal E.L., Moore D.C., Akelman E., Wolfe S.W., Crisco J.J. (2010). Carpal and forearm kinematics during a simulated hammering task. J Hand Surg Am.

